# Environmental Sounds Recognition in Children with Cochlear Implants

**DOI:** 10.1371/journal.pone.0066100

**Published:** 2013-06-20

**Authors:** Shu-Yu Liu, Tien-Chen Liu, Ya-Ling Teng, Li-Ang Lee, Te-Jen Lai, Che-Ming Wu

**Affiliations:** 1 Institute of Medicine, Chung Shan Medical University, Taichung, Taiwan; 2 School of Speech-Language Pathology and Audiology, Chung Shan Medical University, Taichung, Taiwan; 3 Department of Otolaryngology, National Taiwan University Hospital, Taipei, Taiwan; 4 School of Occupational Therapy, Chung Shan Medical University, Taichung, Taiwan; 5 Department of Otolaryngology, Chang-Gung Memorial Hospital, Chang-Gung University, Linkou, Taiwan; 6 Department of Psychiatry, Chung Shan Medical University Hospital, Taichung, Taiwan; University of Montreal, Canada

## Abstract

The aims of this study were (1) to document the recognition performance of environmental sounds (ESs) in Mandarin-speaking children with cochlear implants (CIs) and to analyze the possible associated factors with the ESs recognition; (2) to examine the relationship between perception of ESs and receptive vocabulary level; and (3) to explore the acoustic factors relevant to perceptual outcomes of daily ESs in pediatric CI users. Forty-seven prelingually deafened children between ages 4 to 10 years participated in this study. They were divided into pre-school (group A: age 4–6) and school-age (group B: age 7 to 10) groups. Sound Effects Recognition Test (SERT) and the Chinese version of the revised Peabody Picture Vocabulary Test (PPVT-R) were used to assess the auditory perception ability. The average correct percentage of SERT was 61.2% in the preschool group and 72.3% in the older group. There was no significant difference between the two groups. The ESs recognition performance of children with CIs was poorer than that of their hearing peers (90% in average). No correlation existed between ESs recognition and receptive vocabulary comprehension. Two predictive factors: pre-implantation residual hearing and duration of CI usage were found to be associated with recognition performance of daily-encountered ESs. Acoustically, sounds with distinct temporal patterning were easier to identify for children with CIs. In conclusion, we have demonstrated that ESs recognition is not easy for children with CIs and a low correlation existed between linguistic sounds and ESs recognition in these subjects. Recognition ability of ESs in children with CIs can only be achieved by natural exposure to daily-encountered auditory stimuli if sounds other than speech stimuli were less emphasized in routine verbal/oral habilitation program. Therefore, task-specific measures other than speech materials can be helpful to capture the full profile of auditory perceptual progress after implantation.

## Introduction

The major role of the human auditory system is to inform the listeners about the presence of occurring auditory signals in the surrounding world. These signals can be perceived as two categories: speech and non-speech sounds (e.g. music, environmental sounds), both have tremendous importance in our daily lives. Perception of daily environmental sounds (ESs) allows individuals to feel safe and connected to the dynamic environment that surrounds them [1]. Children with severe to profound hearing loss may impede not only their perceptual ability of speech signals, but also nonlinguistic auditory signals. Cochlear implantation (CI) has been proven to be a regular and efficient treatment option for deaf persons, and a wide range of sound sources is supposed to be accessible by using more advanced devices, such as music [2] and other non-verbal sounds encountered in daily life.

For decades, the literature on speech, language, and communication outcomes following pediatric implantation in deaf children are extensive, and the resulting benefits in these fields have been confirmed in western as well as in Asian countries [3]–[9]. However, previous studies concerning the auditory performance of identifying daily ESs among children with CI are limited. Several reasons have been proposed to explain why the ES perception was less emphasized in deaf children [10]. One is that few testing materials are available to evaluate ES recognition performance, the other reason is that identification of sounds existed in living environment may be regarded as relatively easier listening tasks compared to linguistic ones, or seem to possess less communicative cues. Actually, the perceptual benefits of ESs after implantation are one of the major advantages reported by the parents of hearing-impaired children with cochlear implants [11]. Indeed, timely confirmation on ES recognition can be valuable for parents as well as for clinicians during programming and counseling. It provides consolation for parents when their children are at the stage of preverbal period or at low-verbal stage. Most importantly, children may feel safer and more alert to interact with their living environment if they can better perceive sounds around them.

Human brain regions involved in recognizing ESs have been identified [12], and distinct cortical pathways are responsible to process different categories of ESs [13]. In addition, the perception of ESs may be similar to speech signals in some respects and different in others [14]. Unlike the origins of speech sounds are always derived from the vocal tract of a speaker, environmental sounds are originated from various sources or events, such as the nature, animals, tools etc., although certain acoustic properties can be overlapped considerably between these two categories of sounds. Empirical data obtained from adults with CIs demonstrated an apparently positive correlation exists between the recognition performance of ESs and linguistic sounds [15], [16]. However, the relationships between speech and non-speech sounds recognition among children with CIs remains unclear and less studied.

The outcome measures on ESs perception among adult patients have been reviewed and summarized in Table 1. As it shows, the subjects included in these studies were mostly postlingually deafened adults, and their recognition ability of daily sounds were only modest (e.g. 45.3% to 56.7% in open-set format). Also, high variability in scores among the subjects was consistently noted. Daily-encountered sounds with distinct temporal-envelope cues and spectral features which are similar to voice-like signals are easier to be perceived for postlingually deafened adults [16], [18]. Conversely, Shafiro's study [15] demonstrated that the acoustic factors only play a limited role in predicting ES identification in adult patients with CIs.

**Table 1:** Summary of previous studies on environmental sound recognition performance by patients who received cochlear implants.

Studies	Material	Subjects	Mean ±SD (range)
Proops et al., 1999 [17]	20 pre-recorded sounds (open-set)	100 adults, mostly postlingual deaf	56.7% (7.5–95%)
Reed & Delhorne, 2005 [18]	40 sounds; 1 in 10 alternatives (closed-set)	11 adults, postlingual deaf	79.2% (45–94%)
Inverso & Limb, 2010 [16]	50 items each list; 144 in total (closed-set and open-set)	22 adults, postlingual deaf	open-set: 48.3%±13.5; closed-set: 71.1%±11.5
Shafiro et al., 2011 [15]	60 sound tokens; 1 in 60 alternatives (nearly open-set)	17 adults, postlingual deaf	45.3±16.2% (16–69%)
Current study, 2012	30 sound tokens; 1 in 4 alternatives (closed-set)	47 children, prelingual deaf	67.6%±22.5

The relationship between ESs recognition performance and the acoustic characteristics of different sound events have been investigated in adult listeners who use CIs. However, this relationship is not yet clarified in children with CIs. Unlike adults with CIs who are mostly acquired deafened and may be familiar with these ESs, congenital deaf children have a relatively limited auditory experience before implantation. ESs entering through their CI devices is totally new to them. So, it is valuable to analyze the possible effect on recognition performance in pediatric patients with ESs possessing different acoustic features.

The major objectives of this study are (1) to document the recognition performance of environmental sounds (ESs) in children with CIs and to explore the possible associated factors (2) to examine the relationship between perception of ESs and receptive vocabulary level (3) to explore the acoustic factors relevant to perceptual outcomes of daily ESs in pediatric CI users.

## Materials and Methods

### Participants

A total of 47 children with CIs (31 males, 16 females), aged from 3 to 10, who met the following inclusion criteria, were included in this study: (1) congenital or prelingual deafness, (2) no additional handicaps (e.g. intellectual disability, attention deficit, autism, etc.), (3) no cochlear anomaly and normal cochleovestibular nerves, (4) used Nucleus 24-channel device (Contour, Freedom & N5) (5) the duration of CI use was longer than 3 months. All participants were further divided into two groups: group A included 21 preschool children aged from 3 to 6 years with a mean age of 4.55 (SD = .59), group B comprised 26 school-aged children aged from 6 to 10 with a mean of 7.62 (SD = 1.30).

The demographic data of children for both groups were shown in Table 2. Four variables were included as independent factors for association analysis: age when aural habilitation began, age at implantation, duration of CI usage, and unaided hearing threshold of the better ear. All participants were implanted with Nucleus 24-channel devices by the same surgeon with full electrode array insertion. They all enrolled at several local early intervention centers or special classes for the deaf in which auditory/oral approach was primarily used and most of the older children attended the mainstream elementary school. The study protocol and written informed consent form were approved by Chang-Gung Memorial Hospital Ethics Committee for Human Studies. All written informed consent forms signed by guardians on the behalf of the minors/children participants involved in our study were obtained before the test procedures.

**Table 2:** The mean values (SD) of independent variables of group A (21 preschool children), group B (26 school-aged children) and total participants (n = 47) in this study.

Independent variables	Group A	Group B	Total
Age at aural habilitation began, months	23.45(12.38)	33.05(17.56)	28.48(15.89)
Age at CI surgery, months	35.53(11.76)	56.80(29.13)	47.30(25.21)
Duration of CI use, months	19.07(9.62)	34.61(22.41)	27.67(19.35)
Unaided hearing threshold at better ear, dB HL	99.13(15.40)	98.42(12.25)	98.70(13.38)

### Test Materials

Two tests were administered to measure the perceptual performance of each participant: the Sound Effect Recognition test (SERT) and the Chinese version of Peabody Picture Vocabulary test-revised (PPVT-R).

#### Sound Effect Recognition Test

The Sound Effect Recognition test (SERT) was developed by Finitzo-Hieber et al. [19] and is commercially available from Auditec Ltd. The test was specially designed to assess the sound recognition ability achieved by children with hearing impairment or children with low-verbal level. The test consists of a colored template and a compact disc which contains one practice item and 30 sound effects. Direct implementation is possible for Taiwanese children, due to the nonlinguistic character of the SERT itself. No translation was needed except the response sheet for scoring purpose. The authors have conducted a validation study of Chinese SERT on 141 normal kindergarten children from 3- to 6-year-old in our country [20]. We found that children with normal hearing can reach over 90% correct in SERT by age 5, in average, which was quite similar to those of American children [21]. The mean score of Chinese preschool children in SERT was 90.54% (SD = 8.83%) and the 90% range was 70 to 100% correct. The cutoff point at 5^th^ percentile (i.e.70% correct) was applied in this study.

#### The Chinese Version of PPVT-R Test

The receptive ability of vocabulary comprehension was measured for each child using the Chinese version of Peabody Picture Vocabulary test-revised (PPVT-R) [22]. The normative values of typical developing children from 3 to 12 years of age were provided by the test developer. A standard score and the percentile value can be derived using an age-matched scale provided in the test manual with a mean of 100 and a standard deviation of 15. Some examples of this test material were shown in the appendix of another paper [7].

### Test procedures

For SERT test, the participant was asked to point to one picture among four alternatives on a colored template after listening to the sound. The sound items were played out through a portable CD player. The loudspeaker of the CD player was placed 45 cm in front of the child. The presentation level was approximately 70 dB SPL monitored by a B & K 2239 sound level meter. A total of 30 sound items from three subtests were delivered to each subject. In addition, the order of three subtests (A, B and C) was randomized. The average test time for the SERT was about 20 minutes.

The procedure of administering the PPVT-R test was similar to that of SERT. After listening to the vocabulary spoken by the examiner, the child was asked to choose by pointing to one picture among four alternatives in a response template. Test procedure will continue until the stop criterion was met. The presentation level was about 65–70 dB SPL, also monitored by the same sound level meter described above. The average test time for the PPVT-R was about 15–20 minutes.

A specially trained examiner and a research assistant performed the SERT and PPVT-R for each child in a quiet room. A short break of 5–10 minutes was allowed between two tests in order to prevent children from fatigue or inattention, especially for younger ones. Since all participants were unilateral CI users, all tests were conducted under unilateral listening condition through the implanted ear. With the help of the parents, participants were asked to use the settings they used for everyday listening, and they were able to adjust the settings of their CI (i.e. program, volume and or sensitivity controls) to their preferred levels.

Acoustic analysis of environmental sounds.

Three components of the acoustic characteristics: temporal pattern, harmonic features, and sound duration of 30 test items in SERT were analyzed using software Cool Edit Pro. Sounds with distinct temporal patterning and harmonic/spectral features were determined by visual inspection of the waveforms and the spectrograph. The sounds with distinct temporal patterning or with harmonic features were labeled as “+”; those sounds without temporal patterning or harmonics was as “−”. In order to clarify the contribution of acoustic factors on sound identification, we further classify 30 sound effects into three categories A, B and C according to the presence of temporal patterning and spectral cues. Sounds in category A were those with apparent temporal and spectral cues; sounds in category B were those only with one acoustic factor (e.g. temporal or spectral information); and sounds in category C were those without temporal and spectral components.

### Statistical analysis

SPSS 15.0 software was used for descriptive statistics, correlation analysis, and regression analysis. A multiple regression model with stepwise method was used to analyze the predictive factors of recognition performance on environmental sounds and receptive language level among participants.

## Results

### Sound perceptual ability

Table 3 shows the results of two test measures in group A, B and total participants. For environmental sound perception, the mean correct percentage of SERT was 61.24 (s.d. = 23.83), 72.73 (s.d. = 20.45) and 67.60 (s.d. = 22.53) for group A, group B and the total participants, respectively. School-aged Children (group B) performed 11.49% better than preschoolers (group A) on SERT scores, but the difference is not statistically significant (*t* = −1.779, df = 45, *p* = 0.082). A total of 42.55% children with CI had a score below 70 percent correct; specifically, 57.14% among preschool group and 30.77% among the school-aged group. With further inspection, the results indicated that the average performance of participants fell into the bottom 5% of the normal-hearing preschoolers (aged four to six).

**Table 3:** The results of SERT and PPVT-R obtained from total participants and subgroups A and B.

Test measures		Total	Group A	Group B	*p* value
SERT (% correct)	mean	67.6	61.24	72.73	0.082
	(SD)	(22.53)	(23.83)	(20.45)	
	range	17 to 97	17 to 93	30 to 97	
PPVT-R	mean	87.68	90.05	85.88	0.443
	(SD)	(17.61)	(20.2)	(15.54)	
	range	55 to 127	55 to 127	55 to 115	

The statistical differences (*p* value) between groups A and B were demonstrated.

**Figure 1:**
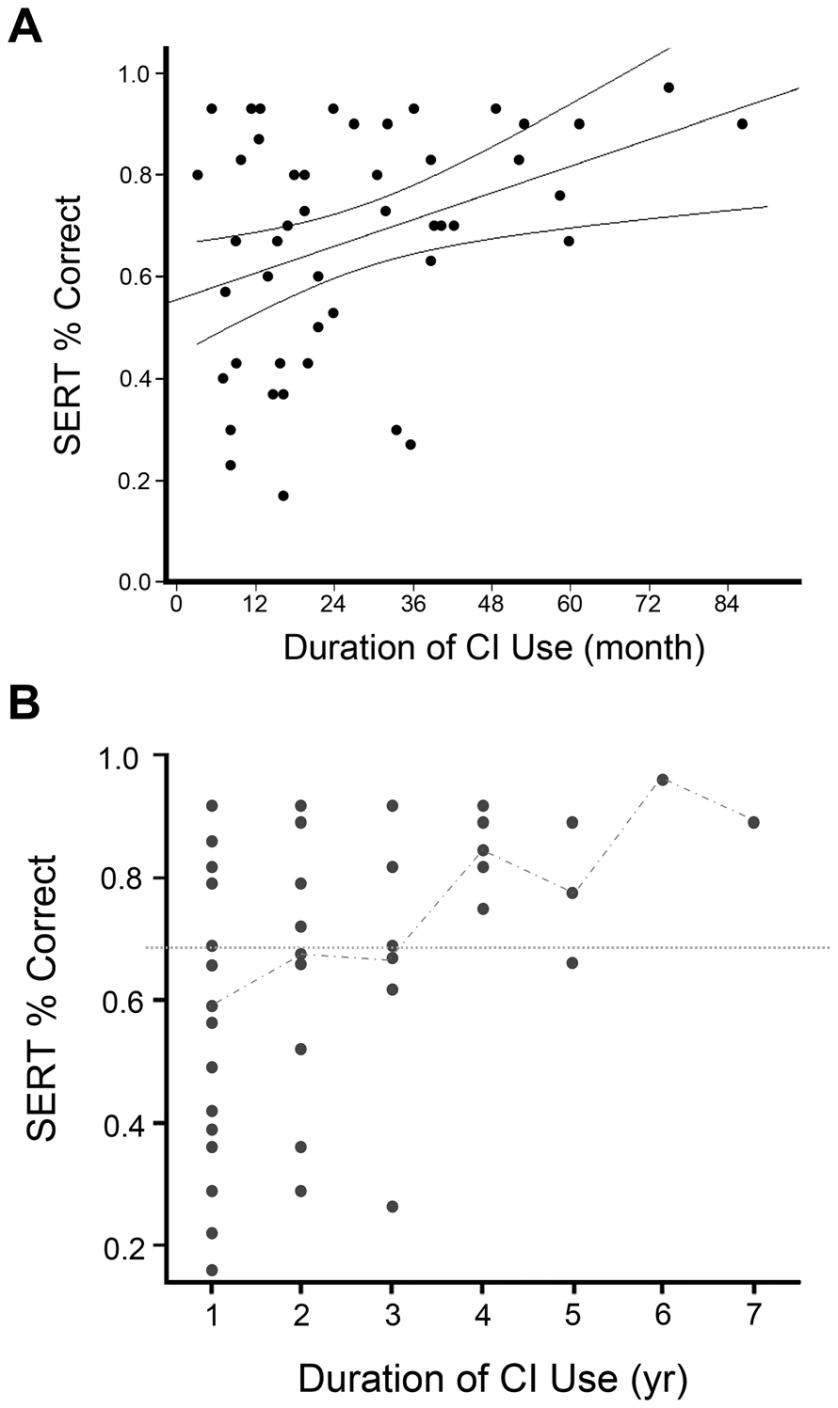
Relationship between correct percentage in SERT and duration of CI use (a). The scatter plot with linear regression prediction line of 95% confidence interval of the predictive factor (i.e. duration of CI use, in months) of correct percentage in SERT. (**b**) Estimated mean values of correct percentage in SERT. The scatter plot with a line of estimated mean values of correct percentage on SERT, the horizontal line across subjects in 7 categories of CI duration was 5% derived from children with normal hearing (5–95% range).

**Figure 2:**
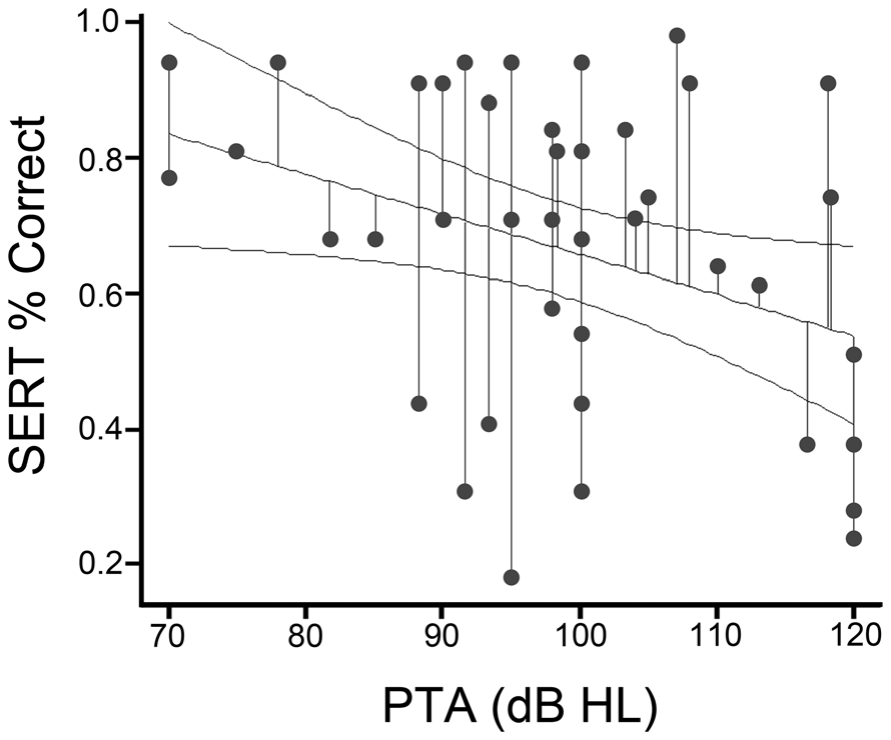
Relationship between the correct percentage in SERT and the PTA average on the better ear. The scatter plot with linear regression prediction line of 95% confidence interval of correct percentage in SERT and the independent variables (i.e.PTA on the better ear, in dB HL).

Two predictors, the “duration of CI use” and “pure tone average”, were associated with the recognition performance on environmental sounds, F (2, 34)  = 7.534, *p* = .002], which accounted for 30.7% of the total variance. The scatter plot with linear regression prediction line of SERT scores (in percent correct) with increasing CI duration is illustrated in Figure 1(a). In addition, the mean values of SERT correct percentage were plotted with increasing duration of CI usage in Figure 1(b). The average correct percentage of SERT was 60.4% by using CI for one year; and reached at least 75% after using CI for four years or longer. A positive correlation was existed between “the duration of CI use” and the correct percentage of SERT, [r = .376, p = .009]. Conversely, the residual hearing level of the participants were negatively correlated with the recognition performance on SERT, the correlation coefficient was r = −.346 (p = .027). Figure 2 illustrates the scatter plot with regression line of the recognition scores of SERT and residual hearing before implantation.

**Figure 3:**
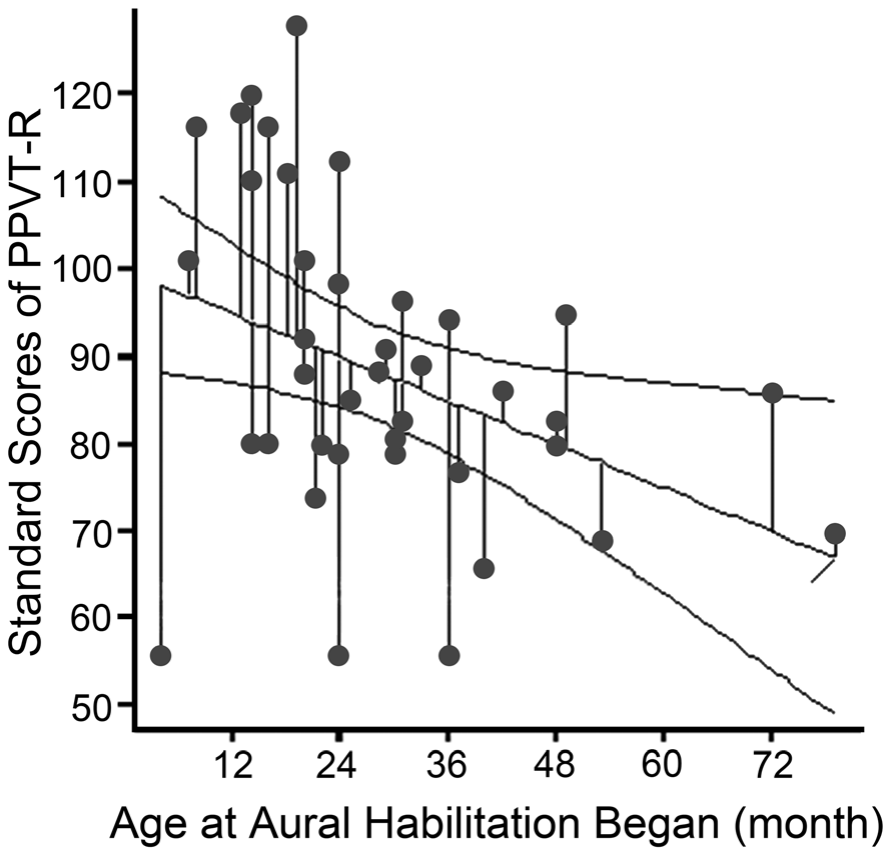
Relationship between the standard scores of PPVT-R and the age at aural habilitation began. The scatter plot with linear regression prediction line of 95% confidence interval of the standard scores of PPVT-R and the age at aural habilitation began (in months).

For receptive language ability, the results shows that the average standard scores of PPVT-R was 87.68 (SD = 17.61) for total participants, 90.05 (SD = 20.20) for children in group A, and 85.88 (SD = 15.54) in group B, respectively. Both groups of children scored at the low average range of normative value. No significant difference existed between the two groups [t = .775, df = 42, *p* = .443]. One p evel [F (1, 32)  = 5.224, *p* = .029], which account for 11.30% of the total variance (Figure 3). There redictor: “age when aural habilitation began”, was associated with the receptive vocabulary l was a negative correlation existed between the standard scores of PPVT-R and “the age when aural habilitation began”[*r* =  −.375, *p* = .019]. In addition, only slight positive correlation existed between the scores of SERT and PPVT-R [r = .335, *p* = .026] (Table 3).

### Acoustic factor analysis

Table 4 shows the results of correct percentage of each sound item recognized by total participants. The acoustic characteristics analysis of each sound item in terms of three acoustic factors (temporal pattern, spectral cue/harmonics, and duration) of sound effects were as follows: 75.30% (SD = 12.42) in category A, 69.33% (SD = 12.07) in was also demonstrated. The average correct recognition scores on three categories category B and 50.51% (SD = 18.45%) in category C. The results of ANOVA analysis showed that the average recognition scores of sound effects in three categories was significantly different from each other [F = 8.132 (2), *p* = .002]. Post Hoc analysis using Bonferroni method showed that sounds in category A were easier to identify than those from category C (*p* = .001); and sounds in category B were easier to identify than those of category C (*p* = .036); however, sounds in category A and B were not significantly different from each other.

**Table 4:** The correct percentage of 30 sound effects in SERT of CI participants listed according to the sound categories (A, B and C, see text).

Category	Sound effects	Correct%	Temporal pattern	Harmonics	Duration (second)
A	dog	100	+	+	5.25
	cat	90.9	+	+	5.8
	baby crying	87.9	+	+	8.77
	drum	84.9	+	+	6.37
	whistle	78.8	+	+	5.47
	bird	78.8	+	+	5.52
	piano	78.8	+	+	10.47
	police whistle	75.8	+	+	4.34
	bells	69.7	+	+	4.46
	telephone	69.7	+	+	7.48
	Woman's voice	66.7	+	+	3.99
	Man's voice	63.6	+	+	3.7
	child sing	60.6	+	+	6.17
	church bell	57.6	+	+	6.41
	Mean (SD)	75.30 (12,42)			
B	hammering	81.8	+	−	8.32
	water splashing	75.8	+	−	6.69
	fire siren	72.7	−	+	15.62
	cough	69.7	+	−	5.9
	clock alarm	63.6	+	−	5.34
	train	54.6	+	−	6.22
	sawing	51.5	+	−	8.94
	Mean (SD)	69.33 (12.07)			
C	gun shot	81.8	−	−	6.12
	door bells	66.7	−	−	5.1
	vacuum	54.6	−	−	9.86
	faucet	57.6	−	−	6.19
	airplane	54.6	−	−	6.5
	toilet	51.5	−	−	9.24
	car start	33.3	−	−	5.77
	dishes breaking	30.3	−	−	6.14
	children playing	24.2	−	−	15.49
	Mean (SD)	50.51 (18.45)			

The notations “+” and “−” represent the presence or absence of the acoustic features (i.e. temporal pattern and harmonics).

## Discussion

Most evaluations of the auditory performance of pediatric CI users have been concerned with speech perceptions. This is the first study which explores the perceptual outcome of children on daily environmental sounds. Our results showed that children with CIs can, in average, recognize approxima.9% in CI adults using close-set testing paradigm. However, only 42.9% of children with CI tely 67.6% of daily-encountered environmental sounds, which is in compatible with 30%–71 performed comparably with preschool children with normal hearing (i.e.>70%), and 30.8% school-aged children with CI (aged from 7–10) still fell behind the normal preschoolers. These results strongly suggest that the ESs perception deserves more attention in pediatric patients after implantation.

Our study also revealed that school-aged participants did not perform significantly better than the preschool participants, and two predictive factors (i.e. “duration of CI use” and “residual hearing before implantation”) were associated with ES recognition abilities. It means that children can recognize more environmental sounds if they use the implants longer, and have more preimplant residual hearing. Furthermore, our data showed that perceptual outcomes improved from approximately 60% correct at the first year after surgery to 80% correct by 4 years' usage of implant devices, suggesting hearing experience play a key role in developing the knowledge and skills required for environmental sound perception, when specific training was not available or limited.

No correlation was noted to exist between recognition ability of environmental sounds and vocabulary language level in our children with CIs. In other words, our children living in the oral-abundant environment cannot get enough experience of non-linguistic sounds and the ability to recognize speech stimuli may not be generalized to the non-linguistic sounds. It is reasonable for us to speculate that sounds other than verbal stimulations may be less emphasized in our auditory habilitation training program. Consequently, these children may need to gain experience by using the implant longer, or by learning these sounds before implantation with the residual hearing.

In this study, no correlation existed between recognition ability of non-speech and speech sounds in our children with CIs. This finding is inconsistent with the positive correlation observed in postlingually deafened adults with CIs [15]. Again, the authors proposed that prelingual deaf children with CIs have to build their auditory perceptual ability by learning the meaning of various sound sources in different context. If sounds other than speech stimuli were less emphasized, the ability can only be achieved by natural exposure to auditory stimuli encountered in daily life gradually. On the contrary, a postlingual deaf adult can retrieve pre-existed auditory memory including speech sounds as well as non-speech sounds with the help of implanted devices. However, it is difficult to compare the performance between children and adults with cochlear implants across different studies, because the stimulus set and response format used were not compatible, as shown in Table 1.

The results of acoustic factor analysis demonstrated that sounds with distinct temporal pattern and/or harmonic features (i.e. category A and B) were easier to recognize than those without (i.e. category C) (Table 4). Three easiest sounds to recognize for CI children were: dogs (100%), cats (90.9%) and baby crying (87.9%). These are all voices which are abundant with temporal and harmonic features. Contrarily, three most difficult sounds for children who used CIs were: car start (33.3%), dishes breaking (30.3%), and children playing (24.2%). The former two sound tokens are noise-like, which contain no distinct temporal and harmonic structure, and the last one is a mixture of various sources usually heard at the playground such as screaming, laughing, running.

It seems that acoustic features play a role in perceiving environmental sounds for children with CI, however, there were exceptions. For instance, the average correct percentage of the sound token “gunshot” was quite high (i.e. 81.8%), in spite of lacking temporal and harmonic cues. We speculate that the abrupt explosion from the unique tool “gun” make it highly impressive by most of the young listeners. Conversely, the average response of two other sound tokens (“woman's voice” and “man's voice”) were only modest (i.e. 67% and 64%), although they are rich in temporal and harmonic features. A possible explanation was that these two voices were produced by English speakers, which may elicit confusion for novel listeners born in a Chinese-based language environment. These results support that cultural/linguistic factor may mediate the acoustic effect on the perceptual process of daily-encountered sounds.

Only 42.9% of the preschool participants performed comparably with their age-matched counterparts. This indicated tha ls. Usually, the parents and teachers of deaf children with CI diligently produce a variety of t at least half of the CI children need specific training of sounds other than speech signa speech sounds repeatedly; however, ESs cannot be reproduced by parents easily. As a result, the children hardly learn the sound-meaning association until enough experience accumulated in the living environment. Moreover, it is worthwhile to notice that the response format was a closed-set one in this study, so children may perform even poorer if they are being tested using open-set paradigm, which is more congruent with the listening challenges encountered in real daily life. Further investigation is needed to address this issue.

Recent studies demonstrates the positive effects of training on the identification of degraded environmental sounds and suggest that training effects can generalize to other sounds in normal-hearing listeners [23]–[25]. Recently, we developed a family-oriented, self-paced, computer-based training program named “The Wonders of the Sounds” with an intention of improving the perceptual ability of ESs for hearing-impaired children. The preliminary results of training effect on 21 participants were quite encouraging [26]. 66.7% (14/21) of CI children improve 20% or higher on a new environmental sound test and four of them improved over 40% after receiving a relatively short-term training. This suggests that auditory training program other than speech stimuli can be very helpful for children in the pre-verbal stage or those with low-level of language ability.

The results of this study also suggest that an environmental sound testing is an ideal assessment tool to monitor the progress of deaf children after implantation, not only for those who have desirable language competency, but also for those with limited speech/language proficiency.

## Conclusion

In this study, we have demonstrated that ESs recognition is not easy for children with CIs, and a low correlation existed between linguistic sounds and ESs recognition in these subjects. ESs recognition in these children can only be achieved by natural exposure to auditory stimuli encountered in daily life over time if sounds other than speech stimuli were less emphasized in routine aural/oral rehabilitation program. Therefore, task-specific measures other than speech materials can be helpful to capture the full profile of auditory perceptual progress after implantation.
